# Blood–spinal cord barrier disruption after spinal cord injury: a time-dependent mechanistic review

**DOI:** 10.3389/fncel.2026.1805529

**Published:** 2026-03-19

**Authors:** Zhirui Jiang, Ce Zhang, Zejing Zhao, Bin Ning

**Affiliations:** Medical Integration and Practice Center, Shandong University, Jinan, China

**Keywords:** blood-spinal cord barrier, neuroinflammation, spinal cord injury, therapeutic targets, timeline

## Abstract

The blood–spinal cord barrier (BSCB) is a specialized vascular interface that preserves spinal cord homeostasis by regulating molecular and cellular trafficking between blood and neural tissue. Disruption of BSCB integrity is a critical pathological event follow-ing spinal cord injury (SCI), leading to increased permeability, inflammatory cell infil-tration, and secondary neurodegeneration. Increasing evidence indicates that BSCB breakdown is not a single event but a dynamic, time-dependent process. In this review, we summarize the molecular and cellular mechanisms responsible for BSCB disruption after SCI in a chronological manner. Key pathological events occurring during the acute, subacute, and chronic phases are discussed, including pathological hemody-namic changes, endothelial stress responses, epigenetic regulation, inflammatory me-diators, immune cell–endothelial interactions, and extracellular matrix remodeling. We further highlight endogenous protective and reparative mechanisms that emerge at later stages. A comprehensive understanding of the temporal characteristics of BSCB disruption may facilitate the development of phase-specific therapeutic strate-gies aimed at preserving barrier integrity, limiting secondary injury, and improving neurological recovery after SCI. This temporal perspective underscores the need for stage-specific interventions to preserve BSCB integrity and improve outcomes after SCI.

## 1 Introduction

SCI is a common disease that causes motor dysfunction. For patients, SCI leads to profound physical impairment and psychological distress, while concurrently placing a substantial financial strain on their families. An epidemiological survey in China showed that there were 759,302 survivors of traumatic SCI in the country, with an annual incidence of 66,374 cases (Jiang et al., 2021). In the United States, about 17,000 people suffer from SCI each year, with an average medical cost of $110,000 for the first year for patients with high-level quadriplegia. The characteristic of SCI is the damage to the spinal cord anatomy, resulting in axonal rupture, neuronal degeneration and necrosis, inflammation, myelin loss, and other pathological changes, leading to severe neurological deficits and impairing the quality of life of patients (Elkabes and Nicot, 2014; Vismara et al., 2020).

Mechanical injury during SCI directly damages the spinal cord tissue and BSCB (Lee et al., 2012a; Nie et al., 2024). After SCI, BSCB breakdown permits the entry of blood components into the spinal cord, precipitating secondary injury characterized by local inflammation, tissue edema, petechial hemorrhage, and oxidative stress (Khayrullina et al., 2015; Wu et al., 2014). Neurons and glial cells undergo “programmed death” due to these secondary injuries, resulting in permanent neurological function loss (Santos-Nogueira et al., 2015). Therefore, drugs that target BSCB may reduce the occurrence of cell damage and functional impairment. Maintaining and restoring BSCB is a key goal of post-SCI treatment measures (Jin et al., 2021).

This article introduces the damage to BSCB caused by related events in the acute, subacute, and chronic phases after SCI, according to the time sequence after SCI, including pathological hemodynamic changes, increased endothelin, endoplasmic reticulum stress, fluctuation of the transcription factor BMAL1, demethylase Jmjd3 upregulation, Heme Oxygenase-1 formation, bradykinin activation of its receptor, HMGB1 production, DJ-1 expression increase, MMP8 expression increase, neutrophil trap NET production, endothelial cell and microglia/macrophage communication, tumor necrosis factor elevation, angiogenin stimulation, TRPM7 channel increase, perforin destruction, and beaded filament remodeling after degradation. By targeting and blocking one of the steps in these events, BSCB damage can be reduced.

## 2 Normal structure of BSCB

**Figure 1:**
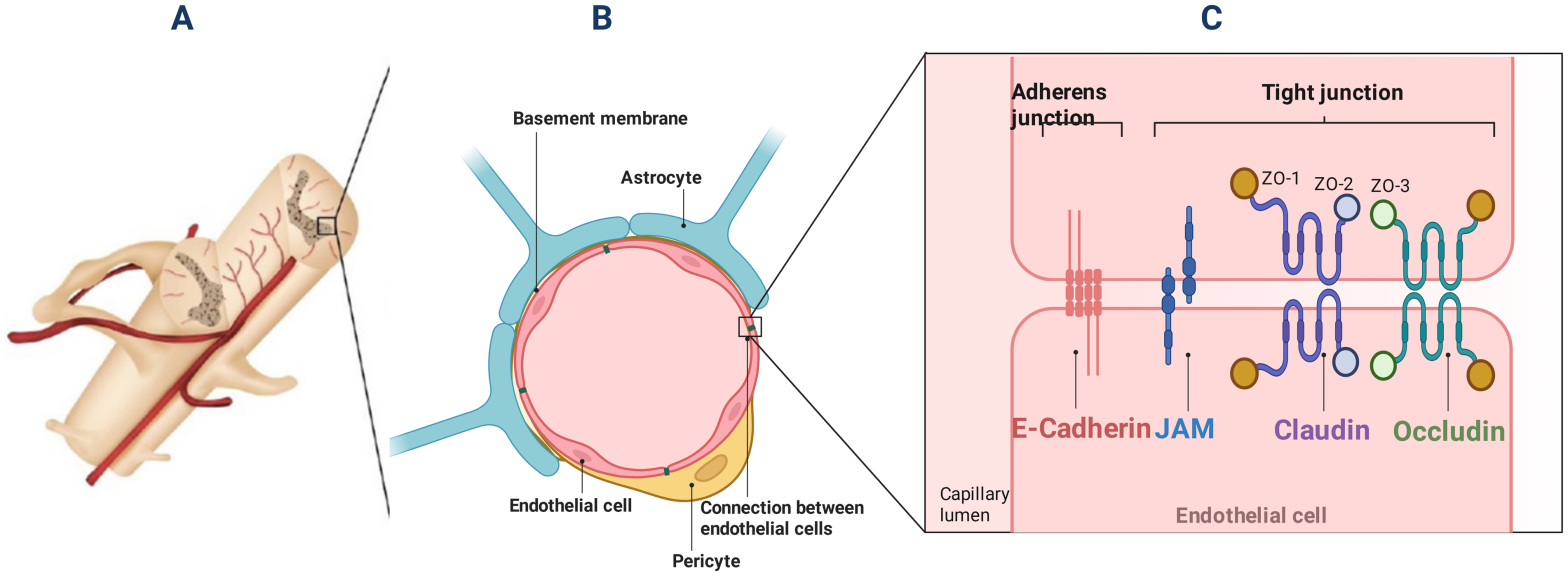
Schematic diagram of BSCB. **(A)** Section of spinal cord and its blood supply; **(B)** Schematic diagram of BSCB cell composition; BSCB is composed of endothelial cells, endothelial cell junctions, basement membrane, pericytes, and astrocytes. BSCB provides a stable microenvironment for the parenchyma of the spinal cord; **(C)** Endothelial cells are bound together by tight junction protein and adhesion junction protein. A typical tight junction consists of occludin, claudin, occludens (ZO-1, −2, and −3) proteins and JAMs. ZO proteins are important for the aggregation of claudin and occludin, leading to the formation of TJ chains; the adhesion protein junction is mainly composed of E-cadherin, which acts as a continuous band on the plasma membrane and allows endothelial cells to have a high level of adhesion to each other.

The structural integrity of BSCB is maintained by endothelial cells with their intercellular junctions, basement membrane, pericytes, and astrocytes. Collectively, these components function to ensure a stable microenvironment within the spinal cord parenchyma (Jin et al., 2025). Endothelial cells are bound together by tight junction protein (TJ) and adhesion junction protein (Figure 1) (Gopal et al., 2025). The following is a detailed description:

### 2.1 Spinal capillary endothelial cells

Spinal cord capillary endothelial cells exhibit distinct morphological and functional features compared to peripheral circulating endothelial cells. These distinguishing characteristics encompass a continuous non-fenestrated plasma membrane, a high mitochondrial density, and an absence of pinocytotic vesicles (Hellenbrand et al., 2021). Endothelial cells contribute to environmental homeostasis within the spinal cord through the regulation of selective transport and metabolic exchange across the blood-spinal cord parenchyma interface (Yao et al., 2023). A critical function of the endothelial barrier is to prevent toxic substances from penetrating the spinal cord parenchyma (Shao et al., 2019). Furthermore, this barrier represents a significant impediment to neurological functional recovery following spinal cord injury (Huang et al., 2024; Yao et al., 2023).

### 2.2 The connection between endothelial cells

The regulation of paracellular permeability through the endothelial barrier is mediated by intercellular junctions. This control relies on the sophisticated molecular organization of junctional proteins and their dynamic interplay with the cytoskeletal framework. The junctional proteins between endothelial cells mainly include tight junctions and adherens junctions (Hawkins and Davis, 2005).

#### 2.2.1 Tight junction proteins

The core constituents of a prototypical tight junction encompass the transmembrane proteins occludin and claudin, the cytoplasmic scaffolding zonula occludens complex (ZO-1, ZO-2, ZO-3), as well as junctional adhesion molecules (JAMs) (Bartanusz et al., 2011; Wang et al., 2023).

#### 2.2.2 Adherens junction proteins

Adherens junctions form a circumferential belt along the plasma membrane, thereby conferring a high degree of intercellular adhesion among endothelial cells (Hawkins and Davis, 2005).

### 2.3 Pericytes

Pericytes establish direct physical contact and maintain signaling communication with endothelial cells via tight junctions, adherens junctions, and gap junctions. These interactions subsequently modulate key endothelial cellular processes, including proliferation, differentiation, permeability, and overall barrier function. Pericytes can secrete various growth factors, inflammatory factors, apoptotic factors, and antioxidant factors, regulating endothelial cell survival, migration, differentiation, and function. Pericytes can also interact with astrocytes, jointly influencing the formation, development, stability, and adaptability of the blood-spinal cord barrier (Bennett and Kim, 2021; Hu et al., 2024; Winkler et al., 2012).

### 2.4 Astrocytes

Astrocytes constitute essential elements of the blood-spinal cord barrier. The terminal endfeet of these cells extensively ensheath the spinal cord vasculature and serve a modulatory function in the expression of endothelial tight junction proteins. This regulatory interaction contributes to the reinforcement of barrier integrity and the restriction of transcellular diffusion (Verkhratsky and Pivoriūnas, 2023). In a manner similar to their role within the blood–brain barrier, astrocytes themselves can establish a distinct barrier. In hypothalamic regions deficient in an endothelial barrier, tight junctions formed between their elongated cell bodies restrict molecular diffusion. Conversely, inflammatory injury to the endothelial barrier can promote the formation of tight junctions among astrocytic endfeet, thereby constituting an emergency astrocyte-derived blood–brain barrier. Whether astrocytes in the blood-spinal cord barrier have similar functions remains to be further investigated (Pivoriūnas and Verkhratsky, 2021; Verkhratsky and Pivoriūnas, 2023).

### 2.5 Basement membrane

The basement membrane (BM), a thin sheet of extracellular matrix molecules, is synthesized and sustained by endothelial cells, pericytes, and astrocytes, contributing to both the formation and preservation of the blood-spinal cord barrier. The BMs are three-dimensional extracellular matrix (ECM) layers composed of four major proteins, including Laminin, Collagen IV, Perlecan, and Nidogens (Thomsen et al., 2017), which form a complex network through interactions, providing structural support to the vessels and regulating endothelial cell proliferation, migration, differentiation, and permeability (Kadry et al., 2020).

## 3 Timeline that causes BSCB to destroy related events after SCI

In pathophysiological terms, SCI is categorized into primary and secondary injury. Temporally, the injury progression can be delineated into the acute phase (within the first 48 h), the subacute phase (spanning from 48 h to 14 days), and the chronic phase (persisting beyond 14 days) (Ahuja et al., 2017; Ribeiro et al., 2023).

### 3.1 Acute phase (<48 h)

#### 3.1.1 Time: 5 min

##### 3.1.1.1 Pathological hemodynamic changes

*In vivo* imaging experiments revealed that pathological blood acceleration occurred around 5 min after SCI, followed by blood stasis or clot removal (de Ruiz Almodovar et al., 2025). Blood components began to leak during the same time period. Since endothelial cells on BSCB are directly exposed to blood flow, pathological hemodynamic changes are one of the factors that lead to increased BSCB permeability and subsequent BSCB disruption (Zhou et al., 2023). The mechanism of pathological hemodynamic changes on BSCB mainly involves two aspects:

Initially, pathological alterations in hemodynamics can impose aberrant mechanical stresses on the vascular wall, leading to its structural compromise (Chiu and Chien, 2011). Subsequently, we computed key hemodynamic parameters affecting the endothelium by applying Poiseuille’s law (Bougouin et al., 2024). The pressure gradient serves as the driving force for maintaining blood flow. Concurrently, it generates an outward force on the vascular lumen, termed transmural pressure, which modulates the passive exchange of water-soluble substances across BSCB (Zhou et al., 2023). Elevated intravascular pressure reflects a heightened outward driving force acting upon compromised vessels, which promotes vascular extravasation, impedes the clearance of tissue fluid, and exacerbates tissue edema (Jacob et al., 2016; Leonard et al., 2015). Under pathological hemodynamic conditions following SCI, a marked elevation in the pressure gradient within the dorsal ascending vein (dAV) was observed. This finding suggests a significant increase in the driving force for permeation across BSCB during this phase (Zhou et al., 2023).

Secondly, the vascular wall is subjected to a physical force generated by blood flow, known as shear stress. Within the range of 10–20 dyn/cm^2^, shear stress is crucial for preserving endothelial barrier integrity. Existing research indicates that deviations from physiological shear stress—whether excessively high or low—can adversely affect both the morphology and function of endothelial cells. Such alterations result in compromised intercellular junctions and consequent dysregulation of barrier permeability (Garcia-Polite et al., 2017). Previous studies have calculated the shear stress and shear rate in the vessels based on Newton’s law of internal friction, using a simplified method (Tarbell, 2010). The shear stress in the dAV increased significantly after SCI, and was not affected by TTM treatment. There is evidence that *in vitro*, shear stress above 40 dyn/cm2 can significantly alter junctional structures and reduce the expression levels of TJ proteins (Garcia-Polite et al., 2017). During the rapid and widespread disruption of BSCB after SCI, more than a quarter of the small veins were under shear stress above 40 dyn/cm2, indicating that this adverse frictional force has a wide range of impact.

**Figure 2:**
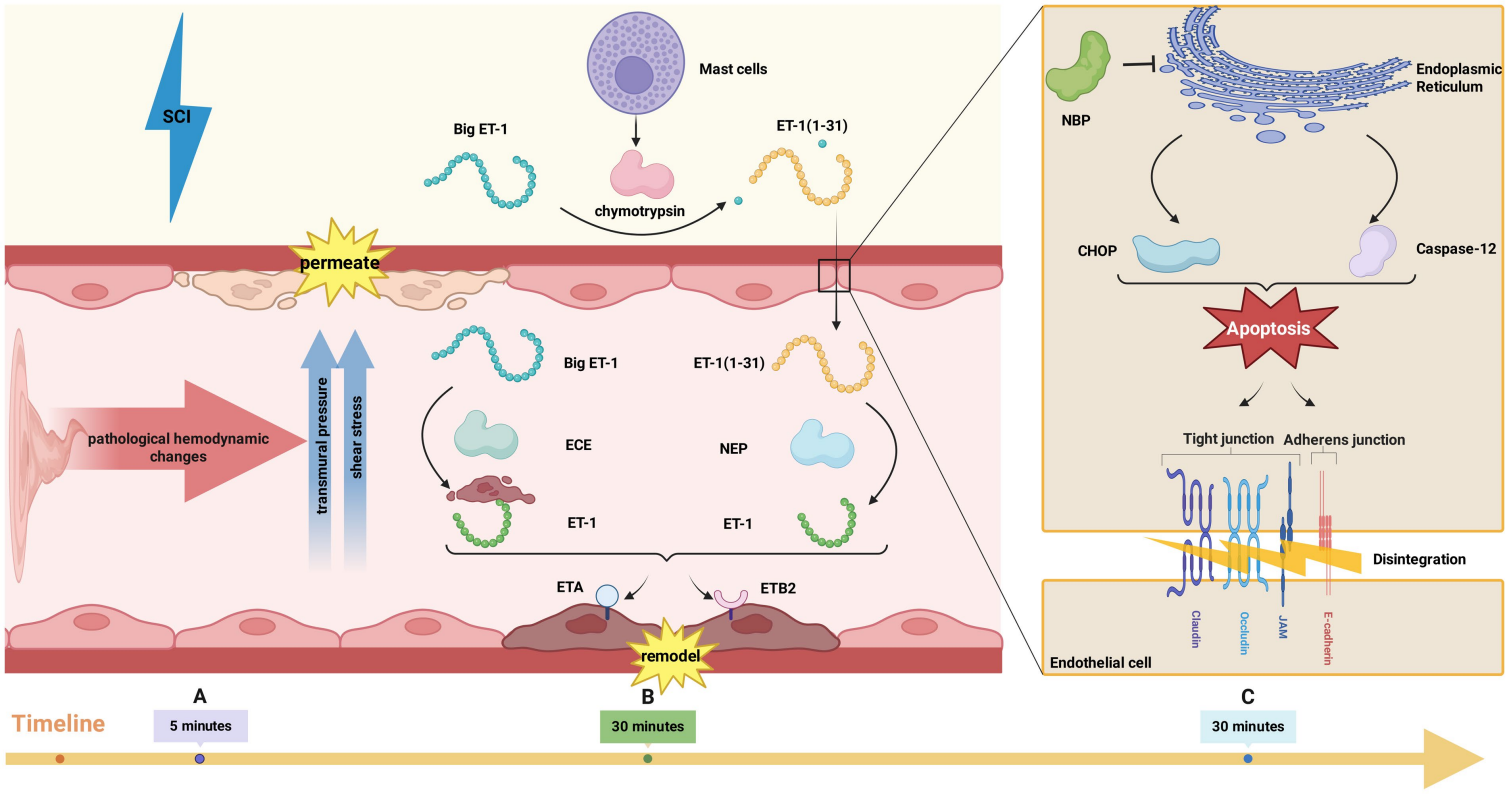
**(A)** Pathological hemodynamics after SCI cause BSCB disruption by applying increased pathological transmural pressure and shear stress to the endothelium; **(B)** Mast cells can secrete chymase, which can convert Big ET-1 into a 31-amino acid intermediate peptide ET-1(1–31). ET-1(1–31) is then hydrolyzed by NEP to produce ET-1. In addition, ECE can directly convert Big ET-1 into ET-1. ET-1 mediates endothelial cell remodeling and causes BSCB disruption through ETA or ETB2 receptor subtypes; **(C)** Endoplasmic reticulum stress after SCI activates CHOP and Caspase-12, which further leads to endothelial cell apoptosis and ultimately results in the degradation of TJ and AJ proteins. NBP can protect BSCB by inhibiting endoplasmic reticulum stress after SCI.

In summary, pathological hemodynamics after SCI lead to BSCB disruption, increased permeability, inflammatory cell extravasation from vessels, and aggravate the severity of secondary injuries such as inflammation. This is achieved by applying increased pathological transmural pressure and shear stress to the endothelium (Zhou et al., 2023) (Figure 2A).

#### 3.1.2 Time: 30 min

##### 3.1.2.1 Increase of endothelin

The concentration of endothelin in spinal cord tissue following moderate injury was measured using an enzyme-linked immunosorbent assay. According to their findings, endothelin levels in the spinal cord exhibited a 50% elevation, which was observed no earlier than 30 min post-injury (McKenzie et al., 1995). Endothelin-1 (ET-1), initially isolated from the culture supernatant of aortic endothelial cells by Yanagisawa et al., is a peptide consisting of 21 amino acids. Following the discovery of ET-1, two further isoforms—endothelin-2 (ET-2) and endothelin-3 (ET-3)—have been characterized. All three isoforms are products of distinct genes, share a common structure of 21 amino acid residues stabilized by two disulfide bonds, and possess six conserved amino acid residues at their carboxyl-terminal ends (Liu et al., 2023).

The endothelin-converting enzyme (ECE) family includes three membrane-bound subtypes: ECE-1, ECE-2, and ECE-3. Among these, the initial two subtypes are capable of being further subdivided into distinct functional variants (Xu et al., 1994). These isoforms exhibit variations at their N-terminal regions and display distinct intracellular localization patterns. The principal role of these enzymes lies in the hydrolysis of the precursor molecule Big ET-1. ECE functions as the key systemic enzyme in the production of ET-1, directly facilitating the maturation of the 38-amino acid precursor into the active peptide (D'Orléans-Juste et al., 2018). The production of endothelin-1 (ET-1) is not exclusively dependent on the ECE-mediated pathway. In embryonic mice with genetic knockout of both ECE-1 and ECE-2, ET-1 levels decreased by only 33% (Yanagisawa et al., 2000). This indicates that there are other pathways to produce ET-1, rather than relying on the typical endothelin converting enzyme. One alternative pathway involves chymase derived from mast cells. This serine protease catalyzes the transformation of Big ET-1 to ET-1. In particular, chymase achieves this by cleaving the Tyr31-Gly32 bond within the 38-amino acid precursor Big ET-1, producing an intermediate 31-amino acid peptide known as ET-1(1–31). Mature ET-1 is then formed both *in vitro* and *in vivo* through the hydrolysis of ET-1(1–31) by neutral endopeptidase, which specifically severs the Trp21-Val22 bond (Fecteau et al., 2005).

The pathophysiological effects mediated by endothelin-1 (ET-1) are partially attributable to its receptor subtypes. At least three such subtypes—ETA, ETB1, and ETB2—are known to influence vascular reactivity within the central nervous system. Among them, the ETA receptor subtype shows a particularly strong association with vascular spasm. The ETB receptor category is subdivided into ETB1 and ETB2. The ETB1 subtype, localized on vascular endothelial cells, primarily mediates vasodilation. In contrast, the ETB2 subtype is expressed on vascular smooth muscle cells and predominantly induces vasoconstriction. Consequently, ET-1-induced damage to spinal cord vasculature, including barrier disruption and vasoconstriction, may be predominantly mediated through the ETA or ETB2 receptor subtypes (Dmour et al., 2023). ETA and ETB receptors are widely distributed within the spinal cord. Their associated signal transduction pathways critically influence inflammation and oxidative stress, both of which are recognized as significant factors impacting neural recovery following BSCB disruption induced by SCI (Ranjan and Gulati, 2022). Within the vascular lumen, the signaling actions of ET-1 are mediated by two specific G protein-coupled receptors: ETA and ETB2 (the latter is not shown). Activation of these receptor pathways facilitates endothelial cell remodeling, upregulates adhesion molecule expression, and impairs the integrity of the blood-spinal cord barrier (D'Orléans-Juste et al., 2018) (Figure 2B).

Moreover, injury to the endothelium upregulates ET-1 expression within endothelial cells and activates monocytes to secrete chemokines, including CXCL8. These chemokines facilitate the margination and transendothelial migration of inflammatory cells across the blood–brain barrier. Additionally, macrophages are capable of converting big-ET-1 into ET-1 and simultaneously producing various cytokines and reactive substances. Among these are tumor necrosis factor alpha, interleukin-1, chemokines like CCL5 and CCL2, and reactive oxygen species, all of which are biologically active within the central nervous system (D'Orléans-Juste et al., 2018; McCarron et al., 1993; Schinzari et al., 2024).

##### 3.1.2.2 Endoplasmic reticulum stress

As a principal subcellular organelle of eukaryotic cells, the endoplasmic reticulum (ER) participates in the synthesis and proper folding of secretory and membrane-associated proteins (Kang et al., 2022). This process is critical for maintaining normal cellular function and viability. ER stress denotes a state of disrupted ER homeostasis triggered by diverse exogenous or endogenous stimuli. This disruption results in the accumulation of misfolded or unfolded proteins within the ER lumen, surpassing its intrinsic processing capacity (Zhou et al., 2021). ER stress has been demonstrated to contribute to both the initiation and progression of SCI. This pathological process subsequently results in disruption of BSCB and neuronal death (Hu et al., 2024; Kang et al., 2022; Zheng et al., 2025).

Research indicates that the upregulation of endoplasmic reticulum stress-related gene expression commences within 30 min post-injury and persists for up to 24 h (Li et al., 2021; Piri et al., 2026). After SCI, apoptosis in EC is initiated by the activation of the ER stress-responsive transcription factor C/EBP homologous protein (CHOP) and caspase-12 (Hu et al., 2025). This programmed cell death subsequently promotes the breakdown of proteins constituting TJ and adherens junctions (AJs). Consequently, the integrity of BSCB is compromised, leading to increased permeability. This allows for the infiltration of blood cells, plasma components, and inflammatory factors into the spinal cord parenchyma, thereby contributing to a range of secondary injuries, including inflammatory pathology. N-butylphthalide (NBP) treatment protects BSCB function by inhibiting ER stress after SCI (Zheng et al., 2017) (Figure 2C).

#### 3.1.3 Time: 6 h

##### 3.1.3.1 Fluctuation of the transcription factor BMAL1

The transcription factor BMAL1/ARNTL is an indispensable element within the core clock circuitry, governing the circadian oscillations of gene expression (Slomnicki et al., 2020). ARNTL/BMAL1, a basic helix–loop–helix transcription factor (TF), functions in conjunction with its binding partners CLOCK or NPAS2 to modulate the circadian rhythmicity of gene expression (Wang et al., 2024). The core circadian oscillator pathway, comprising transcription factors such as Nr1d1/2, Dbp1, Cry1/2, and Per1/2, is regulated by BMAL1: CLOCK/NPAS2 heterodimers. Additionally, tissue-specific target genes of these heterodimers contribute to the circadian regulation of metabolic processes, immune/inflammatory responses, and antioxidant defense mechanisms (Early et al., 2018; Koike et al., 2012; Musiek et al., 2013; Oishi et al., 2017; Rey et al., 2011). While BMAL1 is essential for normal circadian function, its expression is dynamically altered following spinal cord injury (SCI). Studies have confirmed the expression of BMAL1 in spinal cord ECs and shown that its expression is significantly upregulated at 6 and 24 h post-SCI (Slomnicki et al., 2020). Interestingly, experimental reduction of this upregulated BMAL1 promotes functional recovery after SCI. The primary mechanism for this benefit appears to be the alleviation of neuroinflammation through the reduction of BSCB damage and hemorrhage.

**Figure 3:**
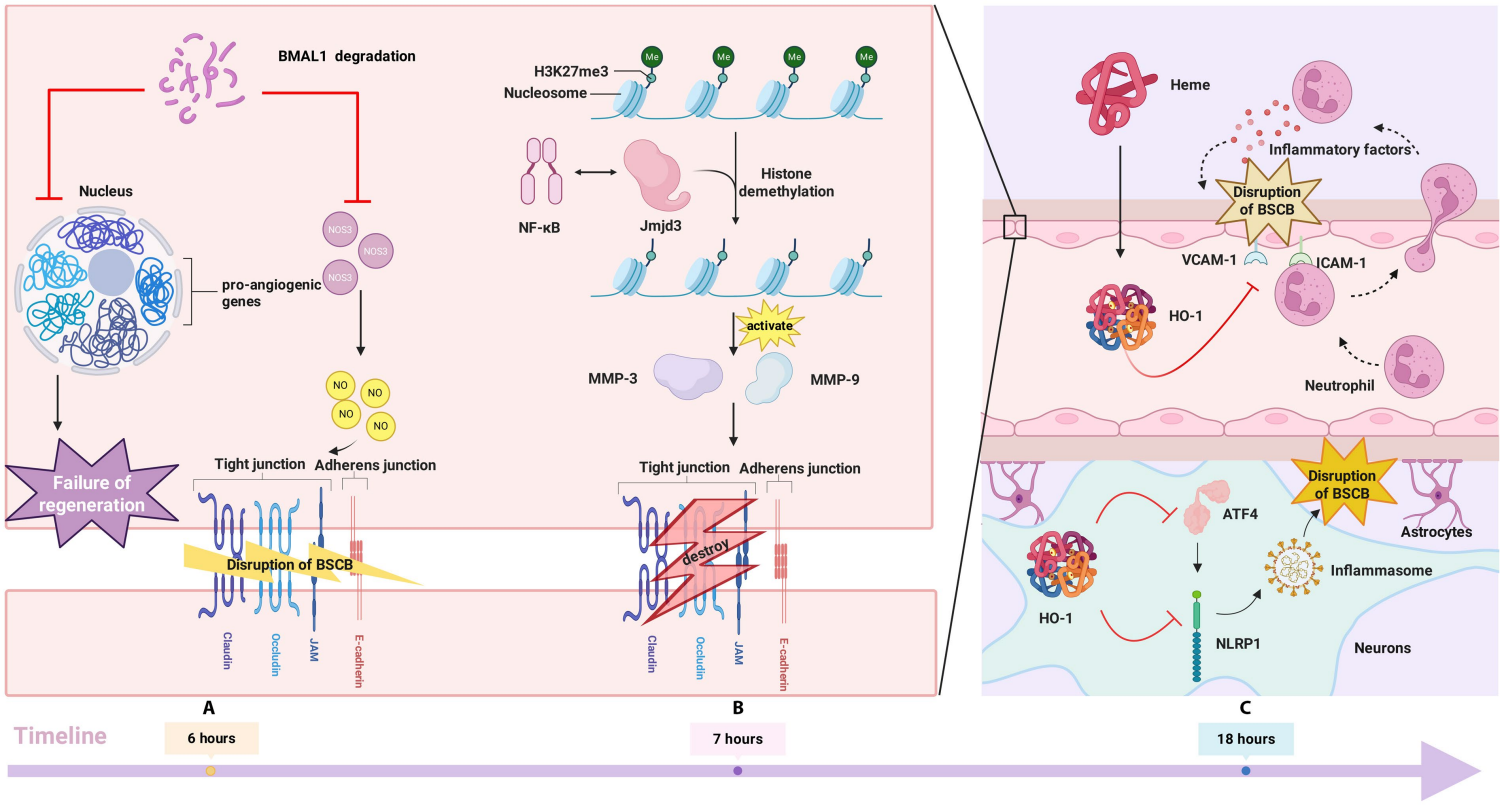
**(A)** BMAL1 degradation reduces NOS3 levels, limiting NO-mediated BSCB damage, and inhibits pro-angiogenic genes, protecting BSCB; **(B)** NF-κB and Jmjd3 interact to demethylate H3K27me3 at *Mmp* promoters, initiating *Mmp*-3 and *Mmp*-9 expression. These degrade TJ proteins, disrupting BSCB; **(C)** Heme leakage through BSCB induces HO-1, limiting vascular adhesion molecules, neutrophil infiltration, and early inflammatory damage. HO-1 directly or via ATF4 inhibition suppresses NLRP1 expression, reducing NLRP1 inflammasome formation and BSCB injury.

BMAL1 reduction alleviates BSCB damage and neuroinflammation through the following mechanisms: Firstly, BMAL1 reduction can decrease Nos3 levels, and Nos3 expression/activity reduction is also beneficial for BSCB function, because NO produced by Nos3 may cause pathological damage to BBB/BSCB (Beauchesne et al., 2009). Furthermore, given the involvement of neovascularization in the delayed deterioration of BSCB function post-SCI, the downregulation of pro-angiogenic genes within the BBB/BSCB module resulting from diminished BMAL1 may represent an additional mechanism for mitigating this delayed functional impairment (Slomnicki et al., 2020). In summary, although BMAL1 is acutely upregulated after SCI, interventions that reduce its levels can decrease BSCB disruption and neuroinflammation. Therefore, BMAL1 represents a potential therapeutic target for mitigating BSCB impairment and attenuating the inflammatory response following SCI (Figure 3A).

#### 3.1.4 Time: 7 h

##### 3.1.4.1 Up-regulation of demethylase Jmjd3

Histone modification represents a significant regulatory mechanism influencing gene expression and associated biological processes, including development, metabolism, pathogenesis, and diverse cellular responses (Bannister and Kouzarides, 2011). Moreover, certain investigations indicate that factors governing histone modifications are critically involved in post-spinal cord injury cellular events, such as glial cell dynamics, neuroprotection, inflammatory regulation, blood-spinal cord barrier disruption, and locomotor function (Lu et al., 2013; Lv et al., 2012). The research indicates that following spinal cord injury, there is an elevation in the levels of Jmjd3, a histone H3K27 demethylase enzyme, within endothelial cells. This increase in Jmjd3 expression occurs within 7 h after SCI (Lee et al., 2012a). Jmjd3 triggers neuroinflammation after SCI through two mechanisms: on the one hand, it activates MMP to damage BSCB; on the other hand, it directly promotes the expression of pro-inflammatory mediators, including iNOS, IL-6, TNF-*α*, COX-2, and IL-1β, in infiltrating inflammatory cells following BSCB disruption, thereby amplifying the neuroinflammatory cascade (Lee et al., 2016). Here are the details:

First of all, Jmjd3 is required to activate Mmp-3 and Mmp-9 genes by demethylating H3K27me3 at the MMP promoter. MMP3 and MMP9 gene activation requires intact NF-κB binding sites. NF-κB functions not only as a transcriptional activator for Jmjd3 but also as a transcription factor that physically interacts with Jmjd3. NF-κB and Jmjd3 cooperate in activating MMP function (Kang et al., 2011). The synergistic increase in Jmjd3 and NF-κB expression is required to initiate the transcription of Mmp-3 and Mmp-9 genes in vascular endothelial cells following injury (Lee et al., 2012a). In summary, Jmjd3 cooperates with NF-κB to promote the transcriptional activation of Mmp-3 and Mmp-9. This is achieved through demethylation of H3K27me3 at the promoters of these Mmp genes. The elevated expression of Mmp-3 and Mmp-9 markedly enhances the permeability of BSCB and contributes to the degradation of tight junction proteins, consequently exacerbating secondary injuries, such as post-SCI inflammation (Lee et al., 2016).

Secondly, Jmjd3 directly upregulates the expression of pro-inflammatory cytokines and enzymes—such as iNOS, IL-6, TNF-α, COX-2, and IL-1β—in immune cells that infiltrate the spinal cord after BSCB breakdown, thus intensifying the local inflammatory response and secondary tissue damage (Lee et al., 2016; Linnerbauer et al., 2020) (Figure 3B).

Furthermore, research indicates that suppressing Jmjd3 expression enhances functional recovery following SCI, while also diminishing axonal degeneration, myelin loss, and the extent of tissue damage. Accumulated evidence indicates that Jmjd3 represents a promising therapeutic target for preventing BSCB disruption following SCI (Lee et al., 2016). Furthermore, the relevance of our results encompasses multiple central nervous system (CNS) disorders associated with BBB/BSCB dysfunction, such as meningitis, epilepsy, Alzheimer’s disease, amyotrophic lateral sclerosis, and multiple sclerosis (Park et al., 2024; Yang and Rosenberg, 2011).

#### 3.1.5 Time: 18 h

##### 3.1.5.1 The formation of heme oxygenase-1

Heme Oxygenase-1 (HO-1) is an enzyme responsible for the degradation of heme, yielding metabolites including carbon monoxide, bilirubin, and free iron. These products exert antioxidant, anti-inflammatory, and neuroprotective actions (Wei et al., 2024). A study showed that the formation of HO-1 after SCI may be induced by the extravasation of heme from the disrupted BSCB. HO-1 induction peaked at 18 h after SCI and decreased at 24 h relative to 18 h. HO-1 limited the damage of inflammation to the BSCB after SCI through two ways. First, HO-1 can alter the expression of vascular adhesion molecules, which regulate the blockade and rolling of neutrophils, so the induction of HO-1 can therefore restrict the migration of inflammatory cells. Thereby attenuating the early infiltration of neutrophils into BSCB and mitigating BSCB damage caused by inflammatory factors (Yamauchi et al., 2004). Secondly, HO-1 has been shown to suppress the assembly of the NLRP1 inflammasome following SCI. As a multi-protein complex, the NLRP1 inflammasome serves to activate both cytochrome c and caspase-1, a process that subsequently induces apoptosis. This apoptotic pathway plays a significant role in modulating neuronal death and inflammatory responses after SCI (Hellenbrand et al., 2021). Experimental findings indicate that the NLRP1 inflammasome is detectable within the cerebrospinal fluid of patients with SCI and may contribute to impairment of BSCB (de Rivero Vaccari et al., 2016). Different types of inflammatory corpuscles not only exist in inflammatory immune cells, but also in neurons. There is increasing evidence to support this notion. It has been demonstrated that NLRP1 inflammasome can induce neuronal damage under high glucose levels (Meng et al., 2014). Activation of Panconnexin channels by high extracellular potassium leads to inflammasome activation in primary neurons and astrocytes (Silverman et al., 2009). Importantly, it should be noted that inflammatory corpuscles have been observed in various types of neurons, including those found in spinal cord injury lesions (de Rivero Vaccari et al., 2012; de Rivero Vaccari et al., 2016; Tan et al., 2015; Walsh et al., 2014). HO-1 can inhibit the formation of NLRP1 inflammasome by down-regulating the expression of NLRP1 in neurons, thus protecting neurons from SCI-induced injury. Furthermore, HO-1 reduces NLRP1 transcription by inhibiting the expression of activating transcription factor 4 (ATF4). This transcription factor, which is upregulated under cellular stress conditions, regulates NLRP1 gene promoter activity and contributes to the increased NLRP1 expression observed after SCI (Lin et al., 2016). Therefore, HO-1 has anti-inflammatory effects in the local area after SCI, reducing the damage of inflammation to the BSCB, thus stabilizing the BSCB (Figure 3C).

#### 3.1.6 Time: 1 day

##### 3.1.6.1 Bradykinin activates its receptor

**Figure 4:**
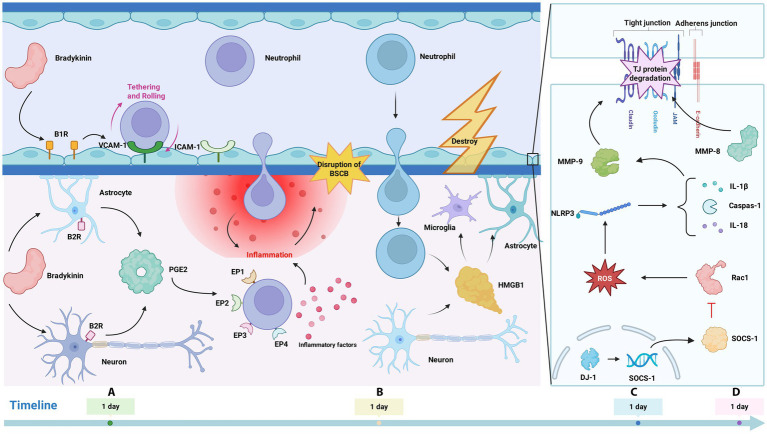
**(A)** Bradykinin activates B1R, upregulating ICAM-1/VCAM-1 to promote leukocyte infiltration and inflammation. Via B2R, it induces neuronal/glial release of PGE_2_, whose receptors (EP1–4) trigger inflammatory factors, damaging BSCB; **(B)** Neuron- and macrophage-derived HMGB1 activates astrocytes and microglia via neural pathways, causing inflammation and BSCB damage; **(C)** Post-SCI DJ-1 upregulation induces SOCS1, inhibiting ROS. ROS activates the NLRP3 inflammasome, promoting maturation/release of IL-18, IL-1β, and caspase-1, creating conditions for MMP-9-mediated TJ degradation; **(D)** MMP-8 can reduce the expression levels of TJ proteins occludin and ZO-1, thereby causing BSCB disruption.

The physiological effects of bradykinin are primarily mediated through its binding to specific receptors on the cell surface. Identified as the two major subtypes, bradykinin receptor B1 (B1R) and bradykinin receptor B2 (B2R) belong to the G protein-coupled receptor (GPCR) family (Astuti and Ysrafil, 2020; Yi et al., 2024). Bradykinin and bradykinin receptors peaked at 1 day after SCI and dropped to the lowest at 7 days after SCI (Yang et al., 2021). Following B1 receptor activation by bradykinin, the expression of leukocyte adhesion molecules, such as ICAM-1 and VCAM-1, is upregulated on endothelial cells. This promotes leukocyte-endothelial interaction, facilitating the migration of leukocytes across BSCB into neural tissue. Within the neural parenchyma, these cells release pro-inflammatory mediators such as TNF-*α*, IL-1β, and IL-6, thereby exacerbating the infiltration of inflammatory cells and factors into the BSCB region (Medeiros et al., 2004; Rex et al., 2022). Bradykinin can also activate B2 receptor, induce neurons and glial cells to release prostaglandin E2 (PGE2), further enhance BSCB disruption and inflammatory response (Brusco and Oliveira, 2025). PGE2 is a cyclooxygenase (COX)-dependent prostaglandin that is synthesized and released in large amounts after SCI. PGE2 exerts its effects by binding to and activating its cognate receptors, EP1 through EP4. This activation modulates the infiltration of inflammatory cells and the release of inflammatory mediators, ultimately intensifying the influx of both inflammatory cells and factors into BSCB (Starikova et al., 2025). In addition, PGE2 can also further induce endothelial cell contraction and tight junction gap formation, reduce BSCB integrity, increase BSCB permeability, facilitate the migration of inflammatory cells from vessels to spinal cord neural tissue (Lee et al., 2020). Therefore, the mechanism of bradykinin in BSCB disruption after SCI is multifaceted, and bradykinin and its receptors may be potential targets for treating BSCB injury and inflammatory response after SCI (Figure 4A).

##### 3.1.6.2 The production of high-mobility group box 1

Tissue analysis from patients with SCI reveals increased expression of High Mobility Group Box 1 (HMGB1). This molecule is implicated in driving neuroinflammation, promoting neuronal death, and facilitating ferroptosis (Kigerl et al., 2018; Ren et al., 2021; Taverna et al., 2022). The HMGB1 protein, consisting of 215 amino acids (aa), is derived from a gene located on chromosome 13q12. The gene structure encompasses 5 exons and 4 introns. Structurally, it contains three domains: two HMG box domains (designated A box and B box) joined by a short flexible linker, and a C-terminal tail characterized by an enrichment of glutamate and aspartate residues spanning approximately 30 aa (Bianchi et al., 1992). The A box domain has HMGB1 antagonistic activity, while the B box has pro-inflammatory activity (Tian et al., 2020). HMGB1 exerts distinct biological functions depending on its subcellular and extracellular localization. This factor can promote the production of pro-inflammatory cytokines, orchestrate cellular activities including proliferation, differentiation, and invasion, and also control autophagic processes (Chen et al., 2022; Yuan et al., 2024).

Following SCI, the upregulation of HMGB1 precedes that of cytokines like TNF-*α*, IL-1β, and IL-6 (Chen et al., 2011). HMGB1 protein levels in the spinal cord show a marked increase within 12 h to 3 days post-injury, peaking on the first day. This release of HMGB1 is primarily mediated via the HMGB1/RAGE or TLR signaling pathways, originating from neurons and macrophages (Sun et al., 2019). HMGB1 is capable of binding to a variety of receptors, including TIM-3, TLR2, TLR4, TLR9, RAGE, and CXCR4. Following SCI, elevated expression levels of HMGB1, RAGE, and TLR2/4 are observed. This increase facilitates the interaction between HMGB1 and either the RAGE or TLR2/4 receptors (Chen et al., 2011). The HMGB1-RAGE pathway can induce macrophage/microglia polarization to pro-inflammatory phenotype, thereby inducing inflammation (Cavalcanti et al., 2025; Fan et al., 2020). The HMGB1/TLR-4 signaling axis also contributes to the activation of inflammatory responses following SCI (Wang et al., 2021). In addition, HMGB1 can also affect the COX2/PGE2 pathway in SCI, leading to astrocyte inflammation and further aggravating the infiltration and damage of inflammatory cells and inflammatory factors to BSCB (Song et al., 2021). Beyond its role in promoting post-spinal cord injury inflammation, HMGB1 can disseminate to the brain via the bloodstream, cerebrospinal fluid (CSF), and axonal transport. This spread contributes to the impairment of both BSCB and BBB. HMGB1 worsens the situation after SCI and hinders SCI recovery, therefore HMGB1 is a potential therapeutic target (Wu and Li, 2023) (Figure 4B).

##### 3.1.6.3 Increased expression of DJ-1

DJ-1 participates in multiple pathophysiological processes, including oncogenesis, the regulation of mitochondrial function, and the inhibition of protein glycosylation (Clements et al., 2006). Prior research has established that DJ-1 exerts neuroprotective effects in the context of neurodegenerative disorders and cerebral ischemia (Wang et al., 2020). A proposed mechanism underlying this protection involves the modulation of inflammatory processes (Zhao et al., 2022). Within the pathological environment of ischemic stroke, DJ-1 demonstrates anti-inflammatory effects through the reduction of pro-inflammatory cytokine levels, such as TNF-α, IL-1β, and IL-18 (Lin et al., 2024; Nakamura et al., 2021; Peng et al., 2020). Conversely, DJ-1 also confers protection against SCI through modulation of BSCB permeability and inflammatory responses. Studies indicate that DJ-1 expression begins to rise within 3 h post-SCI, peaking at 24 h. After 24 h of injury, the protein level of DJ-1 decreased significantly (Cai et al., 2022).

DJ-1 causes BSCB damage and inflammation through the following mechanisms: According to the study, DJ-1 is upstream of SOCS 1 (cytokine signaling suppressor 1), and the elevation of DJ-1 after SCI promotes the production of SOCS 1 (Cai et al., 2022). SOCS1 plays a role in the cellular response to oxidative stress by promoting the degradation of activated Rac1 and inhibiting the production of reactive oxygen species (ROS) (Pedrós et al., 2015). Elevated ROS levels serve as a significant trigger for activating the Nod-like receptor protein 3 (NLRP3)inflammasome (Rubartelli, 2012). Recent evidence further underscores the critical role of NLRP3 inflammasome in driving neuroinflammatory processes (Heneka et al., 2013). Following SCI, the expression of the NLRP3 inflammasome is markedly increased (Luo et al., 2019). Upon activation, it controls the maturation and secretion of key pro-inflammatory cytokines, including IL-18, IL-1β, caspase-1 (Juliana et al., 2012). Within the NOD-like receptor (NLR) family, the NLRP3 inflammasome is predominantly recognized as the archetypal member. Its role is well-documented in the development of neurodegenerative and cerebrovascular diseases (Shippy et al., 2020; Xu et al., 2022). After SCI, NLRP 3 inflammasome was also activated, and blocking NLRP 3 inflammasome activation could alleviate the neuroinflammatory response and improve neural recovery (Jiang et al., 2019). MMP-9, a gelatinase predominantly secreted by infiltrating neutrophils, functions as a key mediator during the early inflammatory phase (Lee et al., 2012b). Following SCI, invading neutrophils release MMP-9, an enzyme capable of degrading the extracellular matrix, tight junction proteins, and other peripheral substrates (Zendedel et al., 2018). Furthermore, activation of the NLRP3 inflammasome initiates downstream signaling cascades and promotes the release of inflammatory cytokines, thereby establishing a pro-inflammatory milieu. This environment subsequently influences the expression levels of both MMP-9 and tight junction proteins, exacerbating the disruption of BSCB (Sozen et al., 2009; Xiang et al., 2025). In summary, DJ-1 blocked the activation of NLRP 3 inflammasome by SOCS 1/Rac 1/ROS pathway, thereby alleviating the damage of blood-spinal cord barrier and related neuroinflammation. DJ-1 is a potential target for treating SCI and deserves further exploration (Cai et al., 2022) (Figure 4C).

##### 3.1.6.4 Increased expression of MMP8

MMPs, a group of ECM proteins, are primarily recognized for their capacity to degrade various ECM constituents. Contemporary studies have greatly expanded the catalog of identified MMP substrates. This extended substrate profile includes diverse molecules such as other proteases, protease inhibitors, coagulation factors, chemokines, growth factor precursors along with their binding proteins, cell surface receptors, intercellular adhesion molecules, and virtually all structural constituents of ECM (Agrawal et al., 2008). MMPs are classified as zinc- and calcium-dependent endopeptidases, with a total of 23 distinct members identified within this family (Page-McCaw et al., 2007). The overall understanding of MMPs is that early inhibition of MMPs can maintain BSCB integrity, reduce cell apoptosis, reduce neuroinflammation and provide early and long-term neuroprotection (Zhang et al., 2011). Following SCI, the expression profiles of MMP family members undergo substantial alterations. These enzymes promote the migration of inflammatory cells into the lesion site and are involved in the initial disruption of BSCB. During their transmigration across the vascular wall, leukocytes release MMPs. These enzymes then degrade proteins associated with tight junctions as well as the adjacent basement membrane (Rosenberg and Yang, 2007). We have already described MMP-3 and MMP-9 damage to BSCB. Recent studies have shown that MMP-8 is also related to BSCB disruption and neuroinflammation after SCI. Here are the details:

MMP-8 expression is highest on day 1 (Kumar et al., 2018). Elevated expression of MMP-8 is observed during the early phase (days 1 and 3) following SCI, suggesting a strong association between MMP-8 and the acute inflammatory stage of SCI. Our observations further indicate that peak MMP-8 levels temporally coincide with maximal neutrophil infiltration and the height of inflammatory activity (Furlan et al., 2025). Given that inflammation represents a hallmark feature of SCI, it is notable that the inflammatory response within the injured human spinal cord demonstrates a high degree of similarity to that observed in rodent models (Donnelly and Popovich, 2008; Fleming et al., 2006).

MMP-8 induces endothelial cell injury through the degradation of TJ proteins. The primary roles of these TJs are to establish both a “barrier” and a “fence” function in BSCB, thereby governing its selective permeability (Bazzoni and Dejana, 2004). TJs are essential for BSCB function regulation, and TJ damage increases permeability and affects BSCB function (Bazzoni and Dejana, 2004; Dejana, 2004). MMP-8 upregulates the expression of TNF-*α*, iNOS, and IL-6 following SCI, while also exacerbating BSCB disruption and cellular injury. One specific mechanism by which MMP-8 impairs the BSCB involves the downregulation of TJ proteins occludin and ZO-1. Structurally, the BSCB is defined by the presence of TJs between adjacent endothelial cells, complemented by a limited degree of transcellular transport. The loss of BSCB integrity and lymphocyte infiltration at the injury site aggravates inflammation, and BSCB rupture after SCI leads to leukocyte infiltration including neutrophils and tissue damage, resulting in secondary injuries including inflammation (Abbott et al., 2006; Bai et al., 2024; Hawkins and Davis, 2005; Kumar et al., 2018; Zlokovic, 2008) (Figure 4D).

### 3.2 Subacute stage (48 h-14 days)

#### 3.2.1 Time: 3 days

##### 3.2.1.1 Neutrophil extracellular trap (NETs) production

Neutrophils represent the initial cohort of inflammatory cells that traverse BSCB and infiltrate the site of injury (Aubé et al., 2014; Carlson et al., 1998; Hsu et al., 2006; Mautes et al., 2000), and they are detrimental to SCI (Feng et al., 2021). After the injury, neutrophils rapidly infiltrate the spinal cord within 1 h, reaching the peak level within 24 h (Carlson et al., 1998; Mautes et al., 2000), and persist in the lesion area for 10 days (Mautes et al., 2000). After migrating to the site of injury, neutrophils generate and secrete various pro-inflammatory mediators. These include oxidases (e.g., myeloperoxidase, MPO), proteolytic enzymes (such as matrix metalloproteinase-9 and elastase), and ROS. Collectively, these released factors play a significant role in inducing secondary tissue injury and worsening neurological impairment (Kolaczkowska and Kubes, 2013; Yates et al., 2021). In addition to releasing cytotoxic products, it has been recently discovered that neutrophils cause various diseases by releasing NET [a type of extracellular fibrous network first reported by Brinkmann et al. (2004), Manda-Handzlik and Demkow (2019), and Morishima et al. (2024)].

Following SCI, infiltrating neutrophils generate NETs. NETs formation peaks approximately three days post-injury and subsequently drives neuroinflammatory responses and compromises the integrity of BSCB (Feng et al., 2021). These early pathophysiological alterations are predominantly characterized by local neuroinflammation and BSCB impairment. These two processes engage in reciprocal interactions, ultimately contributing to the exacerbation of secondary injury following SCI (Ahuja et al., 2017; Alizadeh et al., 2019). Preventing neuroinflammation and BSCB damage is a key measure to interrupt persistent secondary damage (Cox et al., 2015; Kumar et al., 2017). Research indicates that diminishing NETs mitigates neuroinflammation and BSCB disruption within the injured spinal cord region, ultimately fostering tissue repair and advancing the recovery of neurological functions (Feng et al., 2021).

**Figure 5:**
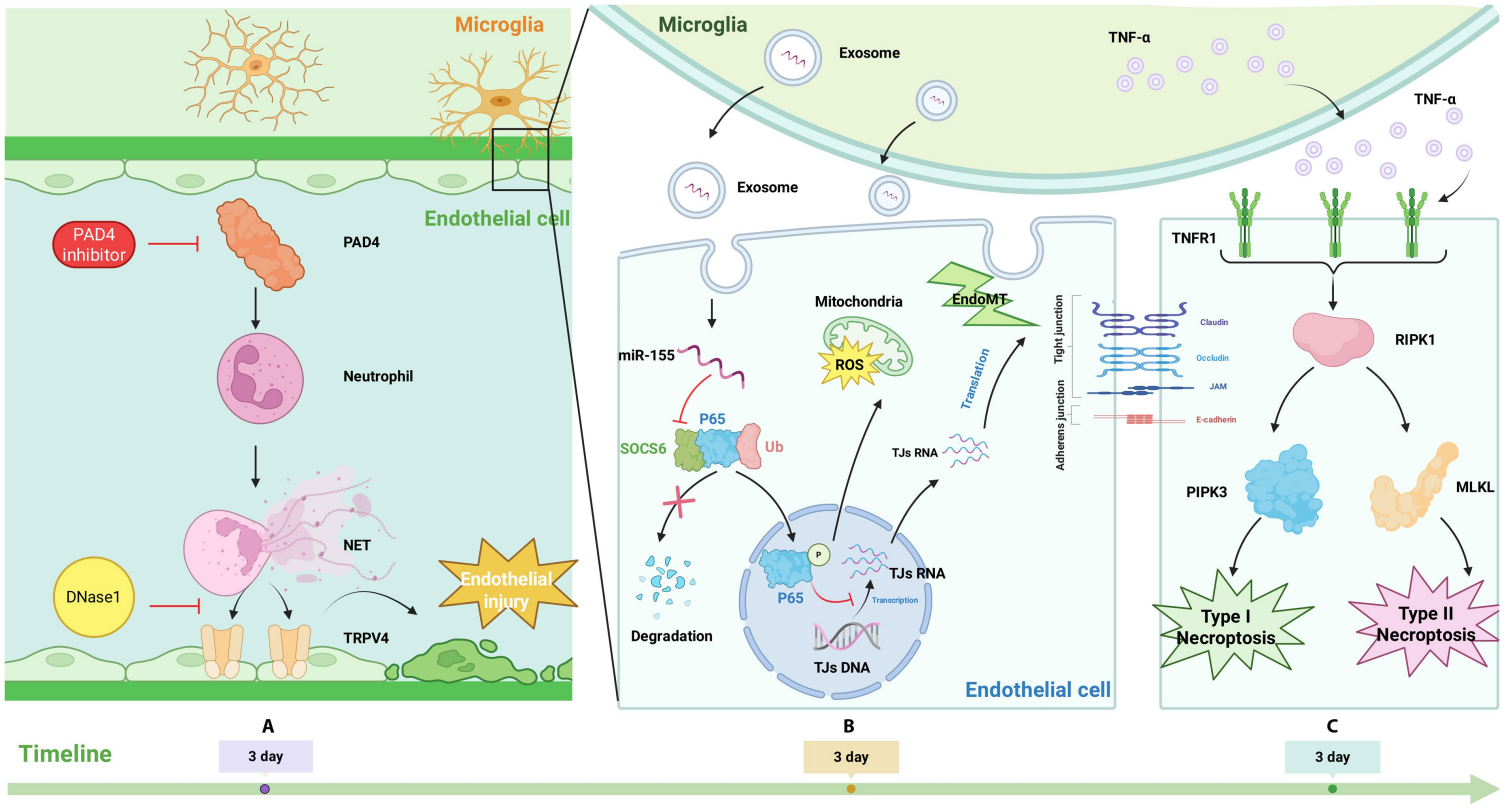
**(A)** Neutrophils cause endothelial cell injury by releasing NETs, which increase TRPV4 in endothelial cells. PAD4 inhibitors and DNase1 can reduce the damage of NETs; **(B)** Microglia release miR-155 to endothelial cells via exosome, which can inhibit the ubiquitination and degradation of P65 through SOCS6, thereby leading to increased nuclear translocation of p65, resulting in increased endoMT and ROS in vascular endothelial cells; **(C)** TNF-α interacts with TNFR 1, activating RIPK1, which activates RIPK3 and MLKL through a series of mechanisms, eventually causing endothelial cell type I or II necroptotic apoptosis.

The possible mechanism of NETs aggravating BSCB disruption is as follows: After SCI, NETs induce an increase of TRPV4 (transient receptor potential vanilloid type 4) in endothelial cells, and the non-selective cation channel TRPV4 has been shown to promote endothelial damage and BSCB disruption after SCI (Kumar et al., 2020). Cl-amidine functions as a peptidyl arginine deiminase 4 (PAD4) inhibitor, targeting the central enzyme responsible for NETs formation. Both suppression of NETs generation via PAD4 inhibition and breakdown of existing NETs using DNase 1 have been shown to reduce BSCB disruption and inflammatory responses (Jorch and Kubes, 2017; Kang et al., 2020; Vaibhav et al., 2020). Overall, NETs intensify neuroinflammation and BSCB impairment, potentially worsening secondary injury after SCI through upregulation of TRPV4. Conversely, impeding NETs formation or facilitating their clearance mitigates tissue damage and enhances motor recovery. These findings indicate that NETs represent a promising therapeutic target in the management of SCI (Feng et al., 2021; Tang et al., 2024) (Figure 5A).

##### 3.2.1.2 Communications between endothelial cells and macrophages/microglia

Communications between endothelial cells and macrophages/microglia occur approximately 3 days after SCI (Ge et al., 2021). In communications between endothelial cells and macrophages/microglia, the increase of ROS and the process of endothelial to mesenchymal transition (EndoMT) are key steps in BSCB disruption after SCI, leading to increased permeability and inflammatory infiltration (Wu et al., 2025). The following introduces the communications between endothelial cells and macrophages/microglia that occur in BSCB after SCI, and focuses on ROS and EndoMT (Cha et al., 2025).

First, let me introduce the communications between endothelial cells and macrophages/microglia. After SCI, exosomes from macrophages induce EndoMT in endothelial cells, affecting the mitochondrial function of vascular endothelial cells. Exosomes derived from M1-type macrophages deliver miR-155 to microvascular endothelial cells. Within these cells, miR-155 targets and suppresses SOCS6, leading to diminished ubiquitination and cytoplasmic degradation of p65, which subsequently results in the activation of the NF-κB signaling pathway. The proteasomal degradation of p65 is hindered, and the nuclear translocation of p65 is increased, resulting in an increase of EndoMT and ROS in vascular endothelial cells. The above process is achieved through communications between endothelial cells and macrophages/microglia (Cha et al., 2025; Ge et al., 2021).

Below we introduce ROS and EndoMT separately. The progression of secondary injury following SCI encompasses a variety of molecular pathways, typically categorized into three principal phases: acute, subacute, and chronic stages (Quadri et al., 2020). ROS, which comprise a group of oxidants generated through redox reactions or electron excitation, represent one of the key molecular drivers underlying this pathological process. These molecules are closely related to mitochondria, and ROS accumulation in mitochondria may cause mitochondrial homeostasis imbalance, resulting in energy loss (Sies and Jones, 2020). In the early stage after SCI, the intracellular mitochondrial ROS level at the lesion site shows a cascade-like increase (Slater et al., 2022). Inhibiting the generation of ROS could contribute to the protection of damaged spinal cord tissues by preventing mitochondrial oxidative stress, neuronal apoptosis, and axonal degeneration. Studies indicate that following central nervous system injury, elevated oxygen consumption in vascular endothelial cells leads to increased mitochondrial ROS levels, which compromises BSCB integrity and induces associated neuroinflammatory responses. This occurs because excessive ROS interferes with vascular regulation of inflammation, cellular proliferation, and mitophagy. Consequently, preserving mitochondrial homeostasis is essential for regulating ROS production in vascular endothelial cells after SCI (Schmidt and Quintá, 2023; Yin et al., 2024).

BSCB endothelium differs from highly permeable peripheral capillary endothelium, as the former mainly depends on TJ to preserve its integrity. Brain injury and SCI result in TJ degradation (Lee et al., 2012; Nag et al., 2007; Yeung et al., 2008), and preventing TJ damage helps to mitigate inflammation and edema induced by BSCB dysfunction (Chen et al., 2014). Interestingly, EndoMT plays a vital role in TJ decline. EndoMT is a process of dynamic cell change from endothelium to mesenchyme, involving the loss of endothelial markers such as TJs and the emergence of mesenchymal, fibroblast or stem cell-like markers (Bischoff, 2019). It is noteworthy that EndoMT cells are mainly located around the vessels (Shenoy et al., 2016). EndoMT has an important role in vascular pathology, and inhibiting EndoMT after SCI can effectively protect neonatal vessels and reduce blood-spinal cord barrier damage and related neuroinflammation (Ge et al., 2021).

In summary, within SCI microenvironment, potential mechanisms of intercellular communication exist between vascular endothelial cells and microglia/macrophages. Furthermore, emerging evidence suggests that exosomes originating from vascular endothelial cells represent a promising therapeutic avenue for SCI (Ge et al., 2023) (Figure 5B).

##### 3.2.1.3 Elevation of tumor necrosis factor

After SCI, microglia at the injury site are activated and produce various inflammatory cytokines, including tumor necrosis factor-*α* (TNF-α) (Chen et al., 2022). TNF-α, an inflammatory cytokine, plays a key role in systemic inflammatory responses (Davies et al., 2007). Following SCI, TNF-α concentrations rise markedly, reaching a peak at 3 days and remaining elevated through 14 days post-injury. By day 3 following SCI, widespread necroptosis of endothelial cells is observed. This process coincides with increased expression of phosphorylated mixed lineage kinase domain-like P-MLKL and P-RIPK3, both recognized markers of necroptotic cell death. Administration of infliximab (INF), a monoclonal antibody directed against TNF-α, or NEC-1, markedly attenuates endothelial necroptosis. Notably, most necroptotic endothelial cells are located adjacent to activated microglia. These findings suggest that TNF-α released from microglia triggers endothelial necroptosis and worsens BSCB breakdown after SCI (Chen et al., 2023). This observation aligns with earlier research demonstrating that TNF-α participates in mediating endothelial necroptosis in disorders of CNS (Chen et al., 2019; Huang et al., 2022). In conclusion, microglial-derived TNF-α promotes endothelial necroptosis following SCI, which intensifies BSCB disruption and increases barrier permeability, thereby exacerbating secondary injury processes such as inflammation (Chen et al., 2023; Kim et al., 2024).

The mechanism of TNF-α inducing endothelial cell apoptosis and causing BSCB damage is as follows: Upon spinal cord injury, activation of microglia results in the substantial release of TNF-α (Huang et al., 2025). This cytokine mediates necroptosis in endothelial cells by activating RIP 1/RIP 3/ MLKL signaling pathway. The engagement of TNF-*α* with TNF receptor 1 (TNFR 1) promotes trimerization and consequent activation of the receptor. Following this, recruitment of RIPK 1 to the intracellular region of the activated TNFR 1 complex occurs. After being activated, RIPK1 activates RIPK3 and MLKL through a series of mechanisms, resulting in type I or II necroptosis of endothelial cells. This destroys BSCB, leading to the occurrence of secondary damage including neuroinflammation (Kim et al., 2024; Lee et al., 2023; Yuan et al., 2019) (Figure 5C).

#### 3.2.2 Time: 5 or 7 days

##### 3.2.2.1 Excitatory effect of angiopoietin

Angiopoietins family has four members: In humans, Ang-1, Ang-2, and Ang-4 are expressed. Notably, Ang-3, which is expressed in mice, serves as the direct murine homolog of human Ang-4 (Lacoste et al., 2025). Ang-2 rises from the first to fifth day after SCI, reaches the highest level on the fifth day, and then drops suddenly from the fifth to seventh day. Ang-1 suddenly rises on the fifth day after SCI, and reaches the highest level on the seventh day (Kumar et al., 2018). Ang-1 enhances the connections between endothelial cells by increasing the expression of key adhesion molecules, notably platelet endothelial cell adhesion molecule-1 (PECAM-1), and TJ proteins such as occludin and ZO-2. This action consequently diminishes the permeability of BSCB (Siddiqui et al., 2015). In addition, Ang-1 can also maintain the stability and integrity of blood vessels by inhibiting the expression of genes related to vascular instability and remodeling, thereby reducing the permeability of BSCB (Nguyen et al., 2020). Reduced permeability of BSCB can inhibit the migration of inflammatory cells from blood vessels to CNS tissues, effectively alleviating the severity of inflammation (Lacoste et al., 2025). Furthermore, Ang-1 is capable of stimulating the phosphoinositide 3-kinase (PI3K)/protein kinase B (Akt) signaling cascade. This activation subsequently suppresses the inflammatory transcription factor NF-κB, leading to a downregulation in the expression of endothelial surface adhesion molecules such as ICAM-1, VCAM-1, and E-selectin. These molecules are critical for facilitating the extravasation of inflammatory cells from the vasculature into sites of tissue damage (Dumas et al., 2016). In summary, Ang-1 can protect the integrity of BSCB and inhibit the infiltration of inflammatory cells into BSCB.

ANG2 competes with ANG1 to inhibit Tie2. Under normal conditions, the agonistic effect of ANG2 can increase the stability and integrity of blood vessels, thereby reducing the permeability of BSCB, which is similar to the effect of ANG1, but weaker than ANG1. Moreover, under inflammatory conditions associated with diminished Tie1 levels, ANG2 acts as an antagonist of Tie2 and triggers a positive feedback mechanism. This mechanism is sustained through foxo1-mediated ANG2 expression, ultimately resulting in vascular remodeling (Lacoste et al., 2025). Therefore, the opposite effect of ANG2 on Tie2 depends on the presence or absence of inflammation (Korhonen et al., 2016). After SCI, due to inflammation, Ang-2 antagonizes the signal of Tie2 receptor, causing the dissociation of TJ and AJ between endothelial cells, and increasing the permeability of BSCB (Benz and Liebner, 2022; Kumar et al., 2017).

**Figure 6:**
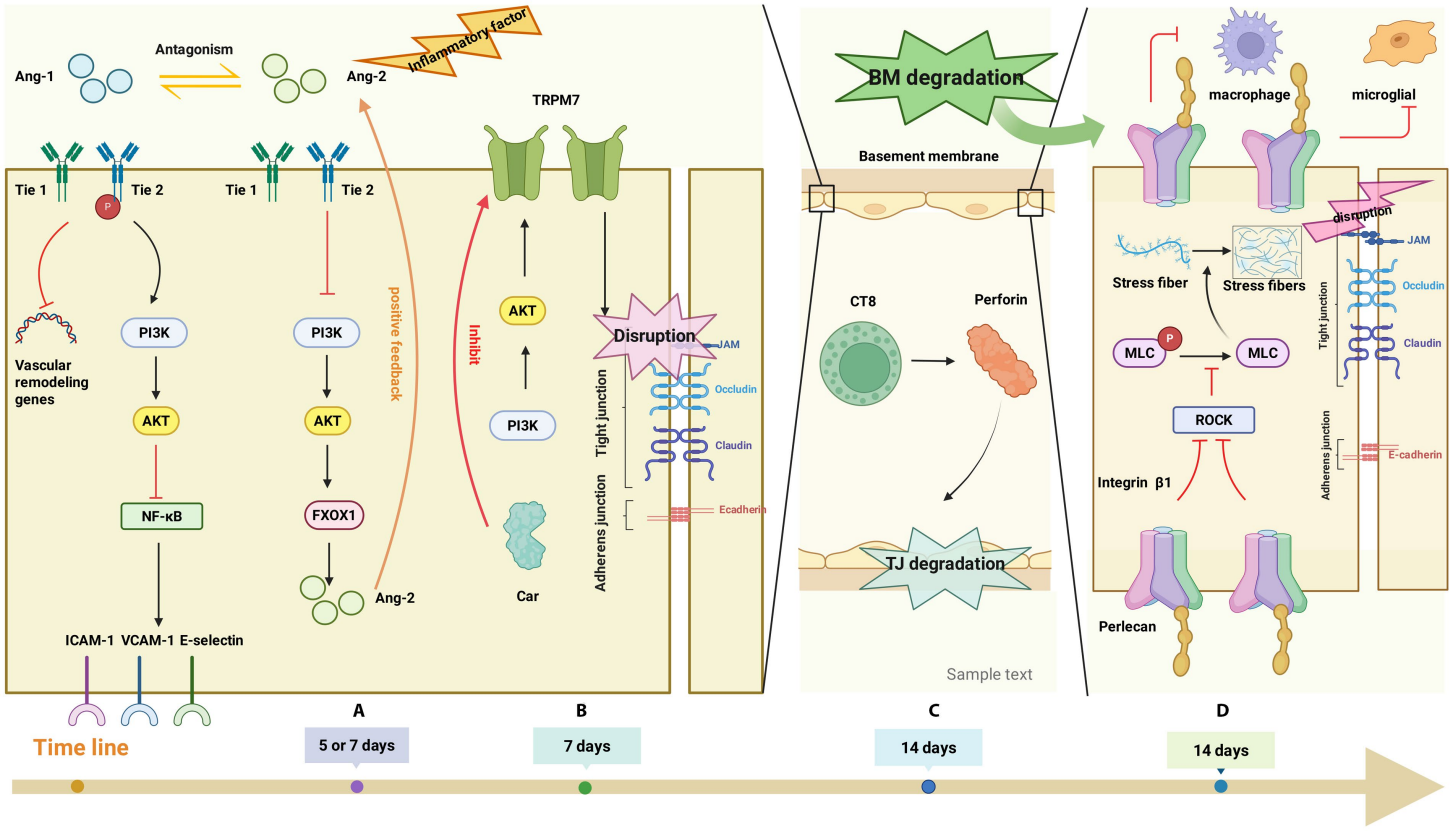
**(A)** Normally, ANG1 activates Tie2 via Tie1, inhibiting vascular remodeling genes and endothelial adhesion molecules. Post-SCI, inflammatory factors reduce Tie1 and ANG2 inhibits Tie2 phosphorylation. This inactivates the PI3K/Akt pathway, promoting FOXO1 transcriptional activity and increasing expression of instability genes (e.g., ANG2). ANG2’s antagonism sustains Tie2 inhibition, forming a positive feedback loop; **(B)** PI-3 K pathway activation upregulates the TRPM7 channel, mediating TJ protein loss. Blocking TRPM7 with CAR reduces BSCB damage and inflammatory cell infiltration; **(C)** Perforin (mainly from CD8 T cells) disrupts TJ proteins, increasing BSCB permeability. This allows inflammatory cytokines from peripheral blood to enter the injury site, exacerbating secondary inflammation; **(D)** Perlecan binds integrin β1, inhibiting the ROCK/MLC pathway. This reduces stress fiber formation and TJ disassembly post-SCI, improving BSCB integrity. Conversely, SCI-induced BM degradation and reduced agrin weaken agrin-integrin β1 binding, promoting stress fibers. Perlecan also inhibits microglia/macrophages.

Angiopoietin-2 (Ang-2) activates endothelial cells by reducing the integrity of BSCB and promotes the extravasation of inflammatory cells from blood vessels, thereby exacerbating the severity of inflammation (Benz and Liebner, 2022, Kumar et al., 2017) (Figure 6A).

#### 3.2.3 Time: 7 days

##### 3.2.3.1 The increase of TRPM7 channel

Transient receptor potential (TRP) channels constitute a family of non-selective cation channels widely expressed across diverse cell types and tissues. For instance, within the nervous system, they are present in neurons and endothelial cells, where they participate critically in numerous cellular functions (Smani et al., 2018; Thakore and Earley, 2019).

Among these, the TRPM7 channel is of particular significance. This channel, which is permeable to metal ions and exhibits non-selective cation channel properties, is ubiquitously expressed across all tissue types. This protein is pivotal in regulating the homeostasis of divalent metal ions, including Mg2+, Ca2+, and Zn2+. It also participates in vital cellular functions such as survival, proliferation, adhesion, and the secretion of neurotransmitters that are dependent on calcium (Inoue et al., 2010; Park et al., 2022). Furthermore, carvacrol (CAR) functions as an inhibitor of TRPM7. By suppressing oxidative damage and modulating the endothelial nitric oxide synthase pathway, CAR confers a protective effect against spinal cord injury (Jiang et al., 2015). Moreover, the blockage of the TRPM7 channel by CAR diminishes blood-spinal cord barrier (BSCB) disruption and the infiltration of inflammatory cells. This inhibition leads to a decrease in the levels of key inflammatory mediators, including TNF-*α*, IL-6, COX-2, iNOS, and chemokines such as Mip-1α, Mip-1β, Mcp-1, Mip-2α, Gro-α, thereby mitigating the inflammatory response. These results suggest that TRPM7 represents a potential therapeutic target for the treatment of spinal cord injury (Park et al., 2022) (Figure 6B).

### 3.3 Medium-chronic injury period (≥ 14 days)

#### 3.3.1 Time: 14 days

##### 3.3.1.1 Destructive effect of perforin

Perforin, an enzyme secreted by NK cells and T lymphocytes, is a key mediator of cytotoxicity and contributes significantly to the pathology of CNS trauma, including SCI (Jakovcevski et al., 2022). Analyses via flow cytometry further revealed that CD8 T cells serve as the primary cellular source of perforin (Shang et al., 2024). After SCI, T cell infiltration peaked at about 14 days, declined for about 2 weeks, and then increased again in the following weeks, reaching a level similar to that at 14 days after injury, and T cell infiltration was still detectable at least 42 days after injury (Hellenbrand et al., 2021; Izadi et al., 2025). Perforin compromises the integrity of BSCB through the disruption of TJ proteins. This disruption facilitates the infiltration of inflammatory cytokines from the peripheral circulation into the lesion site, thereby exacerbating secondary inflammatory responses both within the injured area and its vicinity. In contrast to other tissues, inflammatory damage exhibits greater severity within the CNS. This heightened susceptibility is attributed to the restricted regenerative potential of neurons and glial cells, which often leads to irreversible functional deficits following injury. Consequently, strategies aimed at modulating inflammatory processes represent a promising approach to enhance functional recovery after SCI. Given its involvement in multiple inflammatory pathways—such as the CD8 T cell apoptosis pathway—perforin emerges as a viable candidate for targeted therapeutic intervention (Liu et al., 2019) (Figure 6C).

##### 3.3.1.2 Remodeling of Perlecan after degradation

In the intact spinal cord, Perlecan is localized within BMs. Following SCI, this proteoglycan undergoes significant degradation and subsequent remodeling (Peng et al., 2025). Available data indicate that all cellular components of BSCB, ECs and astrocytes, are responsible for the secretion and maintenance of Perlecan (Soliman et al., 2024; Thomsen et al., 2017). Research has demonstrated that SCI leads to a downregulation in Perlecan expression and initiates its degradation as early as 12 h post-injury. Our observations revealed the recruitment of microglia/macrophages to sites of Perlecan degradation at this 12-h time point. By day 3 post-SCI, co-localization with Iba1-positive cells was noted, and Perlecan within the injury core exhibited a speckled morphology (Gryka-Marton et al., 2025). These findings suggest that microglia/macrophages are recruited to and engage in phagocytosis of Perlecan degradation products during the initial phase of SCI. Furthermore, temporal analysis showed that Perlecan expression associated with laminin increased at 7 and 14 days following SCI, peaking at the 14-day interval. This pattern signifies that Perlecan participates in active remodeling during the post-traumatic phase of SCI (Sasaki et al., 2025; Xie et al., 2023).

Studies have shown that Perlecan can maintain the integrity of the BSCB (Yao, 2019), thereby reducing the neuroinflammatory response. Perlecan has two mechanisms for the above effects: one mechanism is to reduce the inflammatory/immune cells and inflammatory cytokines. The other mechanism is to reduce the permeability of the BSCB after SCI (Xie et al., 2023). Here are the detailed descriptions of these two mechanisms:

Concerning the primary pathological sequence, existing literature confirms that SCI induces an upregulation of inflammatory cytokines and enhances the mobilization of inflammatory and immune cells. These events occur concurrently with the degradation of BM (Orr and Gensel, 2018). Evidence indicates that Perlecan diminishes the population of both amoeboid (spherical) and ramified (branched) microglia, as well as macrophages. Furthermore, Perlecan administration is associated with a decrease in the expression of CD45, a pan-leukocyte marker. Additionally, in the context of SCI, Perlecan has been shown to lower the levels of pro-inflammatory cytokines, such as IL-2, within the injured spinal cord while concurrently enhancing the production of anti-inflammatory cytokines, including IL-4 and IL-10. In conclusion, Perlecan suppressed the neuroinflammatory cascade. This inhibition prevented the recruitment of inflammatory and immune cells, as well as the trafficking of inflammatory cytokines, to BSCB, thereby blocking their entry into CNS and the subsequent induction of neuroinflammation (Xie et al., 2023).

With respect to the second mechanism, integrins—transmembrane receptors composed of α and β subunits—mediate cellular adhesion and facilitate signal transduction either within cells or with ECM (Baeten and Akassoglou, 2011). Strong evidence indicates that Perlecan maintains blood–brain barrier (BBB) integrity by engaging with integrin subunits α1, α2, α5, and β1 (Nakamura et al., 2019). Following SCI, elevated levels of Perlecan enhance the expression of integrin β1. This upregulation subsequently leads to the inhibition of the downstream ROCK/MLC signaling cascade (Shi et al., 2016). Consequently, the formation of stress fibers—which produce centripetal tension, induce contraction of endothelial actomyosin, and contribute to TJ disassembly—is suppressed (Izawa et al., 2018). Therefore, inhibiting the formation of stress fibers can reduce the TJ breakdown after SCI. The integrity of BSCB is predominantly regulated via the paracellular pathway, which is governed by the dynamic state of TJ between endothelial cells. The TJ modulate the passage of substances, thereby restricting the infiltration of serum proteins and inflammatory mediators across the BSCB (Liu et al., 2015). The reduction of TJ breakdown reduces the permeability of BSCB after SCI, preventing the infiltration of inflammatory cells and inflammatory factors through BSCB into the spinal cord parenchyma (Xie et al., 2023).

In addition, Perlecan can also improve axonal regeneration and motor recovery after SCI (Xie et al., 2023). Perlecan is structurally characterized by five distinct domains, each capable of binding to various biomolecules to modulate cellular signaling processes related to migration, proliferation, and differentiation (Roberts et al., 2012). However, the precise proteases responsible for its cleavage, as well as the specific bioactive fragments generated, which contribute to preserving BSCB integrity, attenuating neuroinflammation, and enhancing axonal regeneration and motor function recovery following SCI, remain to be fully elucidated and warrant further investigation (Figure 6D).

## 4 Discussion

This review synthesizes current evidence to delineate the time-dependent disruption and repair mechanisms of BSCB following SCI. Collectively, available data indicate that BSCB breakdown is not a single catastrophic event, but rather a dynamic, multi-stage pathological process driven by distinct molecular and cellular mechanisms across acute, subacute, and chronic phases. Early mechanical and hemodynamic insults initiate barrier failure, while subsequent inflammatory, epigenetic, and immune-mediated events perpetuate permeability changes and neuroinflammation. Importantly, emerging evidence also highlights endogenous repair and remodeling mechanisms, suggesting a therapeutic window for phase-specific BSCB stabilization (Hellenbrand et al., 2021; Jin et al., 2021).

### 4.1 Acute phase: mechanical stress and endothelial vulnerability

In the acute phase, pathological hemodynamic forces—including elevated transmural pressure and excessive shear stress—exert immediate structural damage on endothelial junctions, thereby precipitating rapid BSCB leakage (Zhou et al., 2023). These findings align with broader cerebrovascular literature demonstrating that non-physiological shear stress destabilizes endothelial tight junctions (Lee et al., 2016). Concurrently, early biochemical mediators such as endothelin-1 amplify vascular constriction and inflammatory signaling, further compromising barrier integrity (D'Orléans-Juste et al., 2018).

Endoplasmic reticulum stress emerges as an additional early driver of endothelial apoptosis and junctional degradation, linking intracellular proteostasis failure to macroscopic barrier disruption (Hu et al., 2024; Zheng et al., 2017). These mechanisms collectively establish a permissive environment for inflammatory infiltration within hours of injury.

### 4.2 Subacute phase: epigenetic regulation and inflammatory amplification

During the subacute phase, epigenetic and transcriptional regulators play pivotal roles in sustaining BSCB dysfunction. Upregulation of the histone demethylase Jmjd3 promotes MMP-3 and MMP-9 expression through NF-κB–dependent pathways, directly degrading tight junction proteins and basement membrane components (Lee et al., 2016). Similarly, circadian regulator BMAL1 modulates endothelial nitric oxide signaling and angiogenic responses, influencing delayed BSCB deterioration (Slomnicki et al., 2020).

Inflammatory mediators—including HMGB1, bradykinin, DJ-1, and MMP-8—act synergistically to exacerbate endothelial injury and immune cell infiltration. Notably, NETs represent a recently recognized mechanism linking innate immunity to BSCB disruption, partially mediated by TRPV4 and TRPM7 channel activation (Feng et al., 2021). These findings underscore the central role of immune–vascular crosstalk in propagating secondary injury.

### 4.3 Chronic phase: immune cytotoxicity and barrier remodeling

In the chronic stage, adaptive immune responses contribute to sustained BSCB impairment. CD8^+^ T cell–derived perforin disrupts endothelial tight junctions, facilitating persistent inflammatory trafficking into the spinal cord (Jakovcevski et al., 2022). In parallel, extracellular matrix remodeling—particularly Perlecan degradation followed by compensatory reassembly—emerges as a critical determinant of long-term barrier restoration (Xie et al., 2023).

These observations suggest that chronic BSCB pathology reflects a balance between ongoing immune-mediated damage and endogenous repair mechanisms, highlighting the importance of therapeutic timing.

### 4.4 Clinical and translational implications

From a translational perspective, the temporal stratification of BSCB-disrupting events offers a framework for phase-specific therapeutic intervention. Early strategies may focus on mitigating mechanical stress, ER stress, and endothelin signaling (Kang et al., 2022; Liu et al., 2023; Zhou et al., 2023), whereas subacute interventions could target epigenetic regulators, inflammasomes, and NET formation (Lee et al., 2023; Yates et al., 2021). In later stages, modulation of adaptive immunity and enhancement of extracellular matrix repair may support long-term neurological recovery (Jakovcevski et al., 2022).

Recent advances in endothelial-targeted nanotherapies and exosome-based approaches further support the feasibility of BSCB-centered treatment paradigms (Nie et al., 2024). However, therapeutic efficacy will likely depend on precise alignment with the evolving pathophysiology of BSCB injury.

## 5 Future directions

Future research should prioritize time-resolved, cell-specific analyses of BSCB pathology in both experimental models and human patients. Well-designed longitudinal studies integrating imaging, molecular biomarkers, and functional outcomes are essential. Moreover, combinatorial therapies targeting multiple BSCB-disruptive pathways may prove more effective than single-target approaches. Advances in spatial transcriptomics and single-cell profiling will further refine our understanding of endothelial–immune interactions across SCI progression.

## 6 Conclusion

This review summarizes the time-dependent mechanisms underlying blood–spinal cord barrier disruption following spinal cord injury. BSCB damage evolves dynamically across acute, subacute, and chronic phases, driven by mechanical stress, endothelial dysfunction, inflammatory amplification, immune-mediated injury, and extracellular matrix remodeling. Importantly, BSCB disruption both initiates and perpetuates secondary neuroinflammation after SCI.

Understanding the temporal characteristics of BSCB pathology highlights the importance of phase-specific therapeutic interventions. Targeting barrier-disruptive events while enhancing endogenous protective mechanisms at appropriate time points may provide an effective strategy to reduce secondary injury and promote functional recovery following spinal cord injury.
